# A multicentric multimodal in vivo microscopy MRI study of bipolar disorder reveals axonal loss and demyelination

**DOI:** 10.1192/j.eurpsy.2022.429

**Published:** 2022-09-01

**Authors:** S. Sarrazin, C. Poupon, I. Uszynski, J.-F. Mangin, M. Polosan, P. Favre, C. Laidi, M.-A. D’Albis, M. Leboyer, P.-M. Lledo, C. Henry, L. Emsell, M. Shakeel, V. Goghari, J. Houenou

**Affiliations:** 1 MSP PASTEUR, -, CHEVILLY LARUE, France; 2 CEA Saclay, Neurospin, Gif sur Yvette, France; 3 GIN, Inserm U836, Grenoble, France; 4 APHP CHU Mondor, Psychiatrie, Créteil, France; 5 Institut Pasteur, Unité Perception Et Mémoire, Paris, France; 6 KU Leuven, Translational Neuropsychiatry, Leuven, Belgium; 7 Hotchkiss Brain Institute, Department Of Psychiatry, Calgary, Canada; 8 University of Toronto Scarborough, Graduate Department Of Psychological Clinical Science, Toronto, Canada

**Keywords:** MRI, White matter, bipolar disorder, microscopy

## Abstract

**Introduction:**

Bipolar disorder has been repeatedly associated with abnormalities of white matter. However, DTI is intrinsically limited and the precise cellular mechanisms that underlie these alterations remains unknown.

**Objectives:**

Our aim was to investigate microscopical characteristics of white matter using MRI in patients with bipolar and healthy controls.

**Methods:**

77 patients and 71 controls from 3 sites had a T1 structural MRI, a multi-shell HARDI MRI and at one site with a T1-weighted VFA-SPGR acquisition, and a T2 MSME acquisition. The volume fraction and the orientation dispersion was extracted using NODDI from DW images in each site. Myelin Water Fraction was extracted in 33 patients and 36 controls to probe myelin characteristics. White matter bundles were reconstructed using deterministic tractography. Statistical analyses were performed after harmonization by the ComBat algorithm and controlled for age, gender and handedness.

**Results:**

We found significant lower axonal density in patients along the short fibers of the left cingulum, the left anterior arcuate and the left inferior fronto-occipital fasciculus. We found lower mean MWF in patients along the short fibers of the right cingulum, the left inferior fronto-occipital fasciculus, the left anterior arcuate and the splenium of the corpus callosum. We found higher mean orientation dispersion in patients only along the left uncinate fasciculus.

**Conclusions:**

We report alterations of limbic and inter-hemispheric white matter tracts in patients with bipolar disorder reflecting axonal loss, demyelination and architecture alterations. These results contribute to better capture the plurality of the mechanisms involved in bipolar disorder that cannot be deciphered with classical diffusion MRI.

**Disclosure:**

No significant relationships.

